# Induction Radiochemotherapy for Esophageal Cancer: Long-Term Outcomes from a Single-Center Study

**DOI:** 10.3390/jcm14020394

**Published:** 2025-01-10

**Authors:** Bartłomiej Strzelec, Piotr Paweł Chmielewski, Renata Taboła

**Affiliations:** 1 2nd Department of General Surgery and Surgical Oncology, Medical University Hospital, 50-556 Wroclaw, Poland; bstrzelec94@interia.pl (B.S.);; 2 Division of Anatomy, Department of Human Morphology and Embryology, Faculty of Medicine, Wroclaw Medical University, 6a Chalubinskiego Street, 50-368 Wroclaw, Poland

**Keywords:** esophageal cancer, definitive chemoradiotherapy, induction chemoradiotherapy, multimodal therapy, squamous cell carcinoma, adenocarcinoma

## Abstract

**Background/Objectives:** The management of esophageal cancer (EC) remains a significant clinical challenge, particularly in optimizing therapeutic strategies for different stages and subgroups. This study assessed the impact of preoperative radiochemotherapy (CRT) on clinical staging and identified subgroups for whom definitive CRT (dCRT) may provide a favorable alternative to surgery. **Methods:** Sixty-one patients with esophageal adenocarcinoma or squamous cell carcinoma were enrolled. Pre-treatment staging included computed tomography, gastroscopy with biopsy, and comprehensive laboratory evaluations. Patients received preoperative CRT following the CROSS or dCRT protocols based on tumor stage. Surgical approaches included staged esophagectomy or single-stage Ivor Lewis procedures. Four patients declined surgery and were treated with dCRT. Postoperative outcomes were evaluated using pTNM classification. Follow-up included imaging and endoscopic surveillance. Statistical analyses assessed changes in staging and factors influencing treatment outcomes. **Results:** CRT significantly reduced T stage across the entire cohort (*p* = 0.0002), with complete pathological response (pT0N0M0) observed in 54.5% of patients following induction CRT (*p* = 0.0001). Male patients demonstrated a significant reduction in T stage (*p* = 0.0008), while a similar trend in females was not significant (*p* = 0.068). Among patients declining surgery, dCRT demonstrated acceptable oncologic control over a mean follow-up of 4 ± 0.79 years. **Conclusions:** Preoperative CRT effectively downstages EC and achieves high rates of response, especially in male patients. Therefore, dCRT may be a viable alternative in selected patients, emphasizing the need for individualized treatment strategies to optimize outcomes. These findings underscore the importance of refining multimodal approaches in EC care.

## 1. Introduction

Esophageal cancer (EC) remains one of the most lethal malignancies worldwide, with a dramatic increase in incidence over recent years [1]. Due to its aggressive nature and compounded by factors such as poor general health and advanced age among affected patients, EC ranks as the sixth leading cause of cancer-related mortality globally, with a five-year survival rate averaging between 15 and 25% [2,3,4,5,6,7]. Given the complexity and multifaceted nature of EC, optimal management requires a multidisciplinary approach, typically combining chemotherapy, radiotherapy, and surgical intervention [8,9]. In selected cases, patients with early-stage disease (T1aN0M0) may be candidates for curative endoscopic treatment [10,11,12].

For patients with squamous cell carcinoma (SCC) or adenocarcinoma (AC) located more than 5 cm proximal to the esophagogastric junction, the standard of care includes induction radiochemotherapy. This is commonly administered following the CROSS protocol (41.3 Gy) or as definitive chemoradiotherapy (dCRT, 52 Gy). Compared to surgery alone or preoperative chemotherapy, this approach significantly improves radical resection rates (92% vs. 69%), which has been shown to enhance both overall survival (OS) and disease-free survival (DFS) [13,14,15,16,17,18,19]. However, this regimen also poses an increased risk of perioperative complications, necessitating careful patient selection.

For patients with advanced disease, such as cT4b, N3, or M1, or those contraindicated for surgery due to high perioperative risk or personal choice, radiochemotherapy alone has demonstrated substantial benefits [10,20,21]. The RTOG 85-01 trial highlights the survival benefits of combined chemoradiotherapy over radiotherapy alone, with notable improvements in median survival (14 months vs. 9 months) and five-year survival rates (27% vs. 0%) [4,22]. For cervical esophageal squamous cell carcinoma, this treatment reduces the need for laryngectomy and has shown promising survival outcomes [23,24].

With advancements in radio- and chemotherapy techniques, dCRT is now considered a definitive treatment for certain patients with early-stage EC who may not derive additional benefit from surgery. However, there is insufficient evidence to support the broad adoption of dCRT as a standalone treatment, as esophagectomy remains a key component of curative therapy in the multidisciplinary management of EC. For patients who might experience heightened risk from surgical intervention, the decision to omit surgery following CRT should be made cautiously, balancing the curative potential against surgical risk and quality-of-life implications [8,25,26,27].

It is worth noting that the effects of radiotherapy on the tumor microenvironment (TME) present an area of emerging interest and potential controversy. The TME is a complex, dynamic, and cancer-orchestrated system comprising tumor cells, cancer stem cells, fibroblasts, immune effectors, and vasculature. It plays a crucial role in tumor progression and metastasis [28]. High-energy radiation, while effective in inducing cancer cell death, may provoke endothelial cell dysfunction, inflammation, and angiogenesis, potentially leading to enhanced tumor cell migration and metastasis [29]. Experimental data have linked radiotherapy to increased expression of vascular endothelial growth factor (VEGF) and activation of pro-angiogenic pathways, underscoring the complex interplay between therapeutic effects and unintended stimulation of tumor-associated vasculature [30]. These findings highlight the dual-edged nature of radiotherapy in EC management, prompting further research into strategies to mitigate its adverse effects while optimizing therapeutic outcomes.

This study aims to evaluate the efficacy of preoperative radiochemotherapy in modifying the clinical stage of EC. We also seek to identify patient subgroups and their maximum clinical stage for whom dCRT may offer a favorable and potentially more beneficial alternative to the standard treatment regimen.

## 2. Materials and Methods

### 2.1. Study Design and Patients

**Figure 1:**
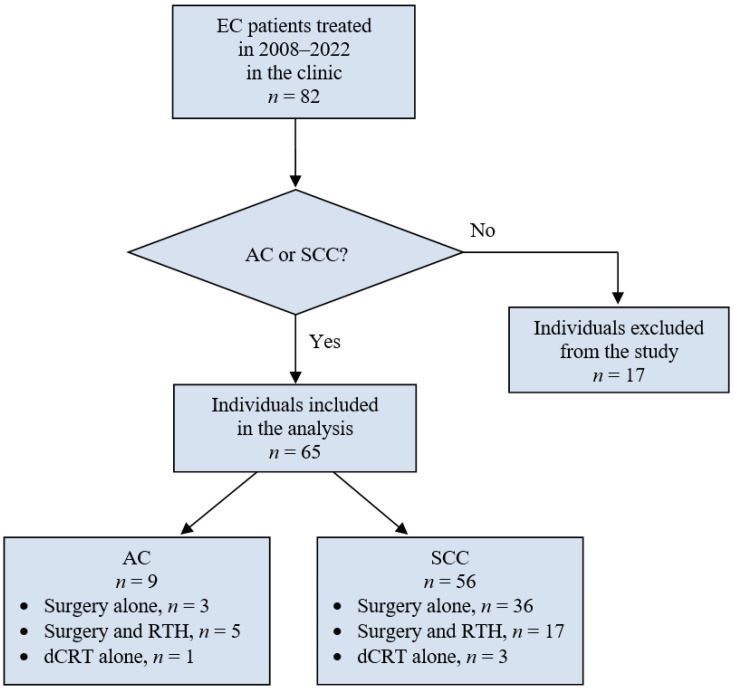
Diagram illustrating the treatment protocol and patient selection process for esophageal cancer (EC).

This study utilized data collected from medical records and histopathological examination results of patients treated for EC at the Department of General and Gastrointestinal Surgery and the 2nd Department of General and Oncological Surgery in Wroclaw from 2008 to 2022. Initially, 82 patients were identified. However, 17 patients were excluded due to rare cancer types (e.g., sarcoma or lipoma), which required different treatment approaches. Consequently, 65 patients with EC, specifically AC and SCC, were included in the analysis. Patient subgroups are detailed in Table 1. Figure 1 depicts the protocol diagram outlining the treatment approaches and the selection process for EC patients.

All patients underwent chest, abdominal, and pelvic computed tomography (CT) to determine clinical staging before treatment. Gastroscopy with lesion biopsy was performed to assess histological grade and tumor type. Baseline laboratory tests included complete blood count with differential, creatinine, urea, C-reactive protein (CRP), electrolytes (sodium, potassium, corrected calcium, magnesium), liver function tests (bilirubin, ALT, AST, GGT, and ALP), pancreatic enzymes (lipase and amylase), total protein, albumin, and coagulation parameters (APTT, INR). Additionally, each patient had a 12-lead electrocardiogram (ECG) prior to surgery and was assessed for general anesthesia suitability by an anesthesiology team. Informed consent for surgery and potential blood transfusions was obtained at least 24 h before the procedure, allowing patients time to address questions and consider their options.

Postoperative clinical staging was assessed according to the pTNM classification by a team of pathologists. Induction radiotherapy (RTH) following the standard protocol was introduced in 2015, with 22 patients receiving induction RTH. The remaining 39 patients were managed with surgery alone. Patients with T2 or lower-stage disease received RTH as per the CROSS protocol (41.3 Gy), while those with T3 or higher-stage disease received RTH as per the dCRT protocol (52 Gy).

For patients undergoing staged treatment, the first stage involved esophagectomy, formation of a salivary fistula, and placement of a nutritional gastrostomy; gastrointestinal reconstruction was considered in a second stage if indicated. Patients receiving single-stage treatment (*n* = 13) underwent the Ivor Lewis procedure. Postoperative follow-up adhered to a standardized protocol, involving alternating CT chest scans and gastroscopies every three months for the first two years, positron emission tomography (PET) after one year, and continued monitoring with alternating CT and gastroscopy every six months for up to five years. Nutritional support was provided through parenteral nutrition following Ivor Lewis surgery and enteral nutrition (via gastrostomy or microjejunostomy) in staged procedures.

Four patients who declined surgical intervention were treated solely with RTH. Of these, three received definitive RTH at 52 Gy, while one received 41.3 Gy as per the CROSS protocol. These patients were followed according to the standard postoperative care protocol, including imaging and laboratory tests.

### 2.2. Statistical Methods

Statistical analyses were performed using the 13.1 Statistica package (StatSoft, Inc., Tulsa, OK, USA). Continuous variables were summarized as means with standard deviations (SD) and medians with interquartile ranges (Q1 and Q3). Normality of distribution was assessed with the *Χ*^2^ test. For comparisons between two groups with normally distributed variables, Student’s *t*-test was applied, whereas the Mann–Whitney *U* test was used for non-normally distributed variables. The *Χ*^2^ test of independence was employed for categorical and dichotomous variables. Logistic regression analysis was conducted to assess the effects of multiple variables, accounting for their interactions, on dichotomous outcomes. Statistical significance was set at *p* ≤ 0.05.

## 3. Results

Changes in clinical staging (cTNM vs. ypTNM) were assessed in all patients who received induction radiochemotherapy (RTH; *n* = 22), focusing on the T and N stages. T downstaging was observed in 20 patients (90.9%). Among these, a two-level T downstaging occurred in 15 patients (68.2%), while a one-level T downstaging was noted in 5 patients (22.7%). No T downstaging was observed in 2 patients (9.1%). N downstaging was documented in 1 patient (4.5%), corresponding to a reduction in one level (N1 → N0). No N downstaging was observed in 4 patients (18.2%), representing 80% of the patients evaluated for N stage. At baseline, 17 patients had an N stage of 0, and they were excluded from N-stage analysis (Not Analyzed, NA) (Table 2 and Table 3).

No statistically significant differences in ypT values were observed based on sex (*p* = 0.213), although cT values approached statistical significance (*p* = 0.045). While a statistically significant reduction in the T stage was not observed in female patients (*p* = 0.068), a significant reduction was evident in male patients (*p* = 0.0008) and across the entire cohort (*p* = 0.0002) (Table 4). A statistically significant reduction in cT-ypT staging was observed following CRT, both for individual T values and overall (*p* = 0.0001). No statistically significant differences were noted in cT values (*p* = 0.099) or in cN-pN changes (*p* = 0.139) (Table 5).

Complete regression to pT0N0M0, defined as the absence of detectable cancer cells in histopathological samples, was observed in 12 patients (54.5%) following induction CRT (*p* = 0.0001). Among the analyzed cases of adenocarcinoma (AC; *n* = 7), five patients underwent induction RTH. Complete regression to pT0N0M0 was achieved in two of these cases, though in the single case with initially positive lymph nodes (N1), no regression was observed following RTH. In two additional cases, the T stage decreased by 1 or 2 (Table 6).

Patients who declined surgery received RTH following the CROSS protocol (41.3 Gy; one patient) or definitive CRT (dCRT; 52 Gy; three patients). This group consisted of three men (75%) and one woman (25%), aged 57–62 years (mean 60 ± 2.55 years), who were monitored for 3.5 to 5 years (mean 4 ± 0.79 years). Detailed data for this subgroup is given in Table 7.

## 4. Discussion

Despite advances in diagnostics, radiotherapy, and surgical techniques, EC continues to rank among the highest causes of cancer-related mortality worldwide [1]. Current evidence underscores the efficacy of multimodal therapy, including radiotherapy, chemotherapy, and surgery, in the management of EC [31]. Due to the disease’s rapid progression and early lymph node involvement, surgical resection with adjuvant therapy remains the principal approach with curative potential [25,32,33,34].

Induction chemoradiotherapy is now recognized as the gold standard for managing both SCC and AC of the esophagus, except in cases involving the esophagogastric junction [14,17]. Our analysis demonstrated that this approach provides substantial clinical benefits, including significant disease downstaging. However, it is also associated with an increased risk of postoperative complications [6,19,35,36,37,38,39,40,41]. These findings are consistent with outcomes reported in previous studies conducted primarily on Asian cohorts. Given the observed impact of chemoradiotherapy on treatment efficacy, it should be a core component of EC treatment whenever feasible. Moreover, dCRT may be a more appropriate option for patients in whom surgical risks are exceptionally high and outweigh the potential benefits [15,24,42].

The observed association between chemoradiotherapy and increased postoperative complications, particularly among patients with no residual tumor cells in surgical specimens, raises important considerations for selecting treatment pathways. This finding suggests a potential benefit for chemoradiotherapy alone, followed by vigilant monitoring, over the standard multimodal approach including surgery in certain patients.

As treatment for locally advanced EC evolves, there is a critical need to integrate personalized approaches that consider tumor biology, patient preferences, and the impact on quality of life [28]. Individualized treatment decisions must also weigh up the potential risks of surgery and long-term outcomes. In this context, dCRT appears particularly promising for patients with early-stage SCC without nodal involvement [43]. In our cohort, all patients with T2N0M0 achieved complete pathological remission (i.e., pT0N0M0) following induction chemoradiotherapy. Although the sample size is modest, precluding definitive recommendations, our findings align with larger studies suggesting that patients with SCC at stages ≤ T1bN0M0 might benefit from a dCRT approach hollowed by active surveillance. This regimen could offer an effective alternative to the standard, more invasive approach, which is often associated with higher perioperative and postoperative risks [35,36,44].

For early stages (T1aN0M0), endoscopic therapy is often effective [11,12]. Thus, patients with T1bN0M0 SCC might represent an optimal population for dCRT as opposed to surgical resection. While two AC patients in our study achieved complete remission without surgery, caution is warranted before extending this approach to AC patients generally. Due to biological distinctions between SCC and AC, the latter of which more closely resembles gastric cancer, surgical treatment remains the standard for AC.

In fact, the TME can be a key factor in determining the varying responses to chemoradiotherapy among patients. As a dynamic and heterogeneous system comprising both non-immune and immune components, the TME can influence tumor resistance to treatment, including CRT. Cancer-associated fibroblasts, immune effector cells, and cytokines within the TME have been implicated in modulating the response to radiation, potentially counteracting the therapeutic effects of CRT [28]. This highlights the need for further research into TME-driven resistance mechanisms and strategies to target these pathways effectively.

Our study also found no significant impact of chemoradiotherapy on N-stage regression, underscoring the importance of surgical intervention for patients with any initial nodal involvement [45,46]. Given that lymph node status is a major predictor of progression-free survival, surgery interventions remain essential for patients with nodal involvement, regardless of tumor histology.

Furthermore, older women, particularly those who are menopausal or post-menopausal, as in this study cohort, represent a unique subgroup due to low estrogen levels, which may influence treatment response. Previous studies have suggested that estrogen modulates the TME and may affect radiosensitivity and immune response in cancer patients, and post-menopausal women may exhibit distinct biological responses to CRT compared to men or pre-menopausal women [47], underscoring the need for tailored therapeutic strategies and further research to understand and address these differences.

For EC patients unwilling to undergo surgery, dCRT offers an important alternative [21,43,48,49]. In our study, none of the CRT-only patients experienced recurrence over a 4.0 ± 0.79-year follow-up. Although our study sample is small and limited to a single institute, our findings suggest that dCRT offers advantages over palliative care alone in patients who decline surgery.

## 5. Conclusions

Overall, this study provides important insights into the management of esophageal cancer in the Polish population, demonstrating that preoperative chemoradiotherapy effectively downstages tumors and achieves high pathological response rates, particularly in male patients. These findings highlight the potential of preoperative chemoradiotherapy to optimize outcomes in carefully selected patients. Additionally, our results suggest that definitive chemoradiotherapy could serve as an alternative treatment in specific cases, emphasizing the importance of personalized treatment strategies. This study underscores the need to refine multimodal approaches in esophageal cancer care to improve therapeutic outcomes.

## Figures and Tables

**Table 1:** Basic characteristics and distribution of patients based on the analyzed feature. All percentages are in bold.

Feature		Women	Men	Total
*n*		19	42	61
**%**		**31.1**	**68.9**	**100**
Age (years)	Mean (±SD)	59.9 (10.3)	60.5 (7.4)	60.3 (8.4)
	Median (Q1, Q3)	60 (57, 66)	60 (56, 66)	60 (57, 66)
Tumor type	AC	0	8	8
		**0**	**13.1**	**13.1**
	SCC	19	34	53
		**31.1**	**55.7**	**86.9**
Surgical Intervention	Ivor Lewis operation	5	8	13
		**8.2**	**13.1**	**21.3**
	Two-stage resection	14	34	48
		**22.9**	**55.7**	**78.7**
Radiotherapy	Yes	4	18	22
		**6.6**	**29.5**	**36.1**
	No	15	24	39
		**25.0**	**39.3**	**63.9**
Resection radicality (Resection margin status)	R0	19	35	54
		**31.1**	**57.4**	**88.5**
	R1	0	7	7
		**0**	**11.5**	**11.5**
Age subgroups (years)	<59	6	16	22
		**9.8**	**26.2**	**36.1**
	59–64	7	11	18
		**11.5**	**18.0**	**29.5**
	>64	6	15	21
		**9.8**	**53.4**	**34.4**
Staging	I	13	15	28
		**21.3**	**25.0**	**46.0**
	II	1	10	11
		**1.6**	**16.4**	**18.0**
	III	5	15	20
		**8.2**	**25.0**	**32.8**
	IV	0	1	1
		**0**	**1.6**	**1.6**
Restaging	0	2	10	12
		**3.3**	**16.4**	**19.7**
	I	12	10	22
		**19.7**	**16.4**	**36.1**
	II	1	6	7
		**1.6**	**24.0**	**11.5**
	III	4	14	18
		**6.6**	**23.0**	**30.0**
	IV	0	2	2
		0	3.3	3.3
Cancer cells found in postoperative specimens	No	2	10	12
		**3.3**	**16.4**	**20.0**
	Yes	17	32	53
		**27.9**	**52.5**	**80.0**
Postoperative complications	No	15	31	46
		**25.0**	**50.8**	**75.4**
	Yes	4	11	15
		**6.6**	**18.0**	**24.6**
Grading	G0	2	10	12
		**3.3**	**16.4**	**19.7**
	G1	4	6	10
		**6.6**	**9.8**	**16.4**
	G2	11	19	30
		**18.0**	**31.1**	**49.2**
	G3F	2	7	9
		**3.3**	**11.5**	**14.8**

**Table 2:** Changes in disease stage before and after the use of induction chemoradiotherapy.

cTNM	ypTNM	Grading	T Downstaging	N Downstaging
T3N0N0	T2N0M0	3	1	NA
T2N1M0	T0N1M0	NA	2	0
T2N0M0	T0N0M0	0	2	NA
T2N0M0	T0N0M0	0	2	NA
T4N1M0	T3N2M0	3	1	0
T2N0M0	T0N0M0	0	2	NA
T2N0M0	T0N0M0	0	2	NA
T3N0M0	T2N0M1	2	1	NA
T4N1M0	T2N0M0	1	2	1
T2N0M0	T0N0M0	0	2	NA
T2N0M0	T0N0M0	0	2	NA
T3N0M0	T1N0M0	2	2	NA
T3N0M0	T2N0M0	0	1	NA
T2N0M0	T0N0M0	0	2	NA
T2N0M0	T0N0M0	0	2	NA
T4N0M0	T2N1M1	2	2	0
T3N0M0	T3N0M0	2	0	NA
T2N0M0	T0N0M0	0	2	NA
T3N1M0	T3N1M0	2	0	0
T3N0M0	T2N0M0	2	1	NA
T2N0M0	T0N0M0	0	2	NA
T2N0M0	T0N0M0	0	2	NA

cTNM, c = the clinical classification of the TNM; T = tumor, N = nodes, M = metastasis; pTNM, p = the pathologic classification of the TNM; yp = pathological TNM classification after RTH; NA = not analyzed.

**Table 3:** Changes in disease stage; the number of patients (*n*) and percentage (%).

Feature	*n*	%
T downstaging	20	90.9
T downstaging by 2	15	68.2
T downstaging by 1	5	22.7
No T downstaging	2	9.1
N downstaging	1	4.5
N downstaging by 1	1	4.5
N upstaging by 1	1	4.5
No N downstaging	4	18.2

**Table 4:** Changes in disease stage cT–pT based on sex. All percentages are in bold.

Feature	Value	Women	Men	Total	Chi^2^ Test, *p*-Value
	*n*	19	42	61	
	%	**31.1**	**68.9**	**100**	
ypT	0	2	10	12	≤0.213
		**3.3**	**16.4**	**19.7**	
	1	6	4	10	
		**9.8**	**6.6**	**16.4**	
	2	6	12	18	
		**9.8**	**19.7**	**29.5**	
	3	4	11	15	
		**6.6**	**18.0**	**24.6**	
	4	1	5	6	
		**1.6**	**8.2**	**9.8**	
cT	1	6	2	8	≤0.045
		**9.8**	**3.3**	**13.1**	
	2	6	19	25	
		**9.8**	**31.1**	**41.0**	
	3	5	14	19	
		**8.2**	**23.0**	**31.1**	
	4	2	6	8	
		**3.3**	**9.8**	**13.1**	
Wilcoxon Matched Pairs Test		*p* ≤ 0.068	*p* ≤ 0.0008	*p* ≤ 0.0002	

yp = pathological TNM after induction RTH; cT = clinical TNM.

**Table 5:** Changes in disease stage cT–ypT and cN–ypN based on the use of induction radiotherapy. All percentages are in bold.

Feature		No CRT	CRT	Total	Chi^2^ Test, *p*-Value
	*n*	39	22	61	
	**%**	**63.9**	**36.1**	**100**	
Cancer cells	No	0	12	12	≤0.000.1
		**0**	**19.7**	**19.7**	
	Yes	39	10	49	
		**63.9**	**16.4**	**80.3**	
pT	0	0	12	12	≤0.000.1
		**0**	**19.7**	**19.7**	
	1	8	2	10	
		**13.1**	**3.3**	**16.4**	
	2	13	5	18	
		**21.3**	**8.2**	**29.5**	
	3	12	3	15	
		**19.7**	**4.9**	**24.6**	
	4	6	0	6	
		**9.8**	**0**	**9.8**	
cT	1	8	0	8	≤0.099
		**13.1**	**0**	**13.1**	
	2	13	12	25	
		**21.3**	**19.7**	**41.0**	
	3	13	6	19	
		**21.3**	**9.8**	**31.8**	
	4	5	3	8	
		**8.2**	**4.9**	**13.1**	
cT–pT	Worsening	1	0	11	≤0.000.1
		**1.6**	**0**	**1.6**	
	No change	38	2	40	
		**62.3**	**3.3**	**65.6**	
	Improvement by 1	0	5	5	
		**0**	**8.2**	**8.2**	
	Improvement by 2	0	15	15	
		**0**	**23.0**	**23.0**	
cN–pN	Worsening	0	1	1	≤0.139
		**0**	**1.6**	**1.6**	
	No change	38	18	56	
		**62.3**	**31.0**	**96.6**	
	Improvement by 1	0	1	1	
		**0**	**1.6**	**1.6**	

pT = pathological classification; cT = clinical classification.

**Table 6:** Changes in stage of esophageal adenocarcinoma (AC) after the use of induction radiotherapy.

TNM	pTNM	Grading	T Downstaging	N Downstaging
T1N0N0	T0N0M0	NA	1	NA
T3N0M0	T3N0M0	2	0	NA
T3N0M0	T2N0M0	2	1	NA
T3N1M0	T3N1M0	2	0	0
T2N0M0	T0N0M0	NA	2	NA

NA = not analyzed.

**Table 7:** Patient characteristics treated exclusively with radiotherapy (RTH).

Sex	Age (Years)	Histological Type	Clinical Stage	Type of Radiotherapy	Follow-Up (Years)
Man	62	SCC	T2N0M0	CROSS	3.5
Man	63	SCC	T3N1M0	dCRT	5
Woman	57	AC	T2N0M0	dCRT	3
Man	58	SCC	T2N0M0	dCRT	4.5

## Data Availability

If required, our data can be submitted.
